# Supplementary material for: Reactogenicity to major tuberculosis antigens absent in BCG is linked to improved protection against Mycobacterium tuberculosis
Source: Nat Commun. 2017 Jul 14;8:16085. doi: 10.1038/ncomms16085 (PMC5519979; doi:10.1038/ncomms16085)
Supplement: Supplementary Information [file ncomms16085-s1.pdf]

File name: Supplementary Information
Description: Supplementary Figures

File name: Peer Review File
Description:

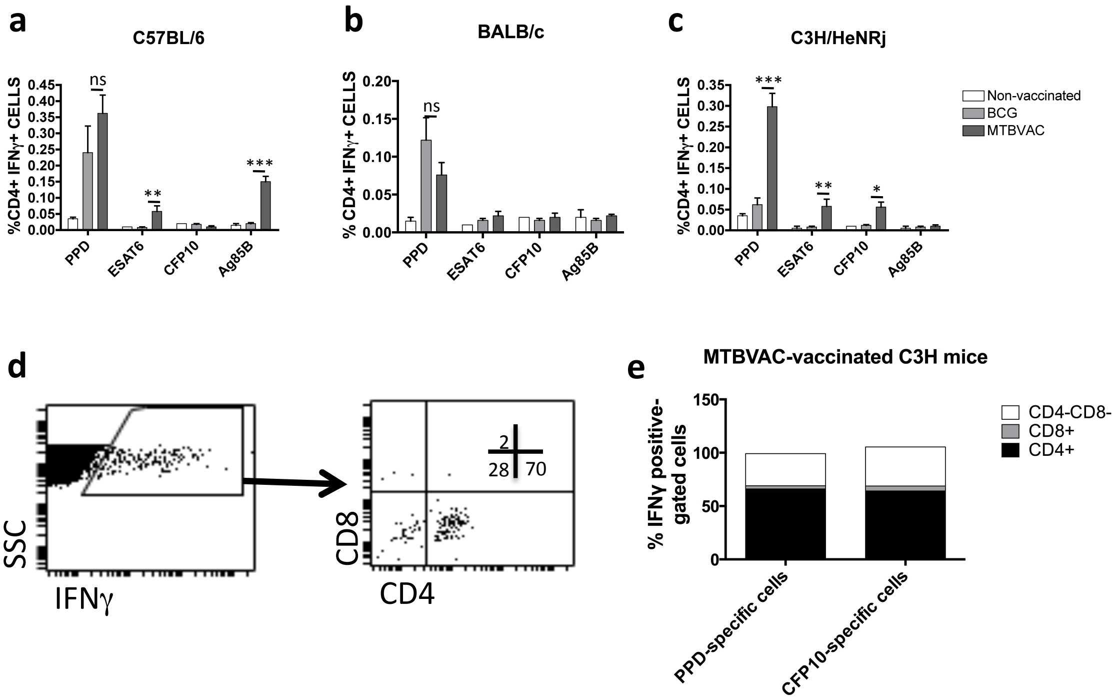

Supplementary Figure 1. Percentage of IFNγ-producing CD4+ splenocytes from mock-, BCG- and MTBVAC- vaccinated C57BL/6 (a), BALB/c (b) and C3H/HeNRj (c) mice following stimulation with PPD (5 μg/ml), ESAT6 (2 μg/ml), CFP10 (2 μg/ml) and Ag85B (2 μg/ml). Data are representative from one of two independent experiments (n=5 mice/group/experiment). All data are mean ± SEM. ns, non-significant; *, $p < 0.05$; **, $p < 0.01$ ***, $p < 0.001$ by unpaired t-student test. (d), (e), CD4+, CD8+ and CD4-CD8- cells were analyzed from an IFNγ-positive gated population of splenocytes harvested from MTBVAC-vaccinated C3H mice. (d) Representative dot-plot is shown in the figure. (e) Data from the graph is the mean from one experiment (n=5).

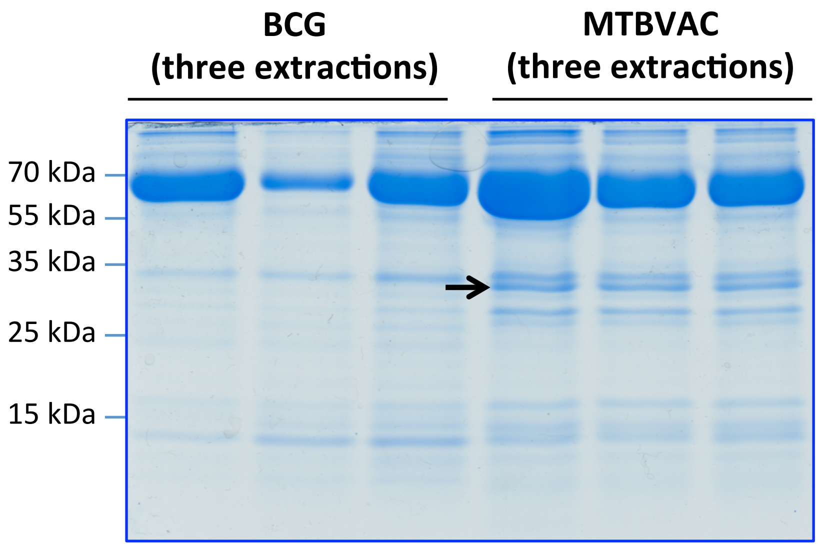

Supplementary Figure 2. Culture-filtrate protein samples from BCG and MTBVAC separated by SDS-PAGE and stained with Colloidal blue. Three independent extractions from each vaccine strain are represented in the image. A differential band found in MTBVAC (pointed with an arrow) was identified as Ag85B by MALDI-TOF MS.

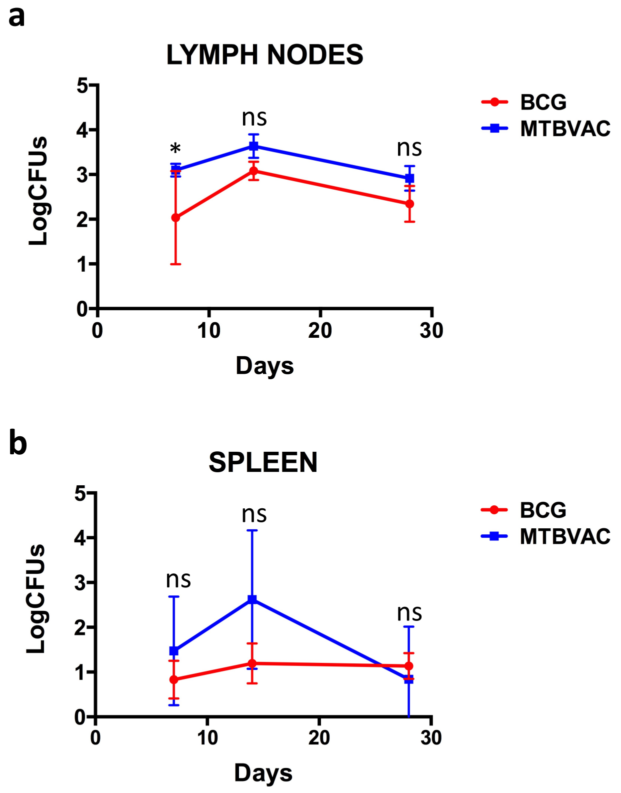

Supplementary Figure 3. Dissemination and replication of MTBVAC and BCG in lymphoid organs from C3H mice. MTBVAC and BCG CFUs in draining lymph nodes (inguinal and axillary) (**a**) and spleen (**b**) at 7, 14 and 28 days following subcutaneous vaccination of C3H in the right rear flank. Data are derived from n=6 mice. All data are mean ± SD. ns, non-significative; *, $p < 0.05$; by two-way ANOVA and Bonferroni post-test.

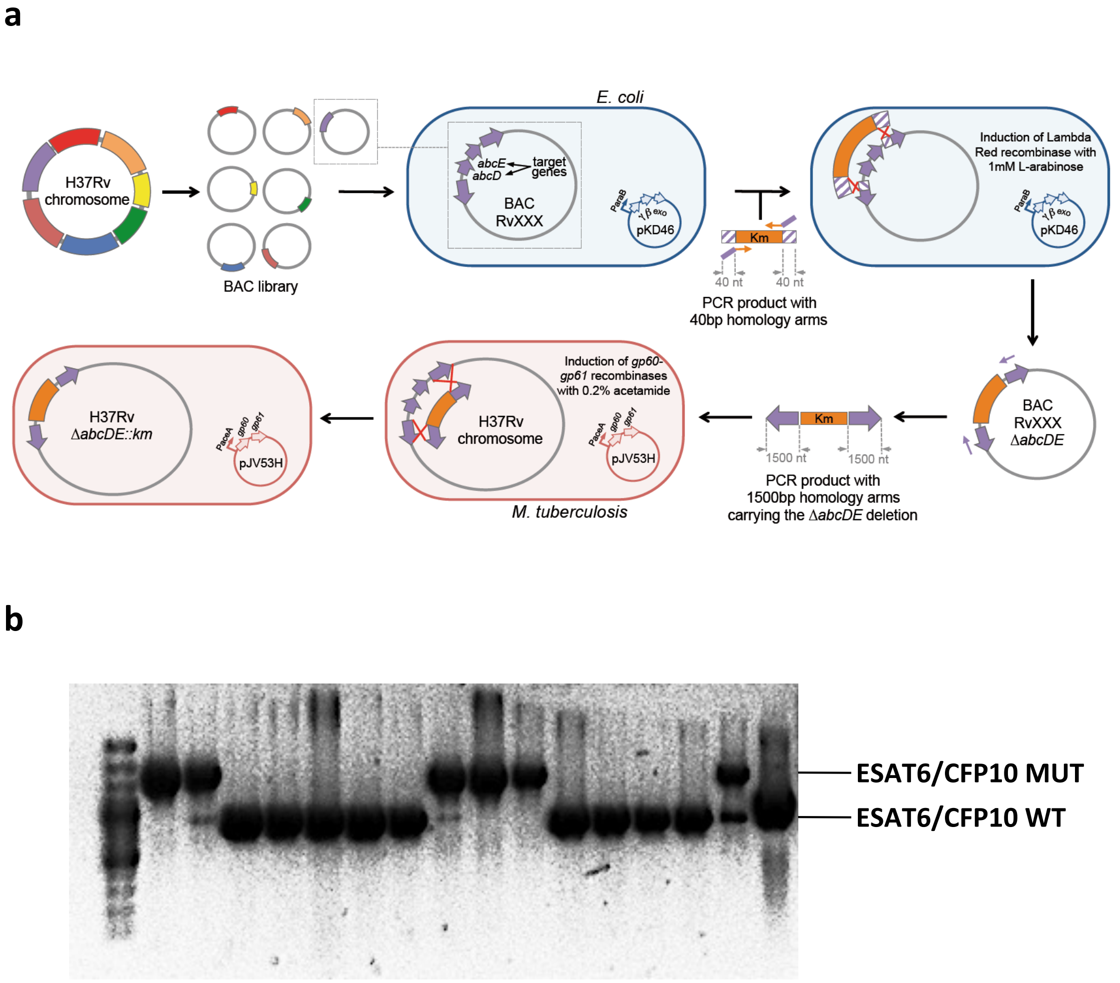

Supplementary Figure 4. Construction of MTBVAC mutant substrains. (**a**) General scheme of the method used to construct the MTBVAC mutants tested in this study. (**b**) Analysis of recombinant MTBVAC colonies after recombineering. Agarose electrophoresis shows CFP10/ESAT6 region amplified with flanking primers. Wild-type amplicon: 0.8 kbp; mutant amplicon: 1.7 kbp.

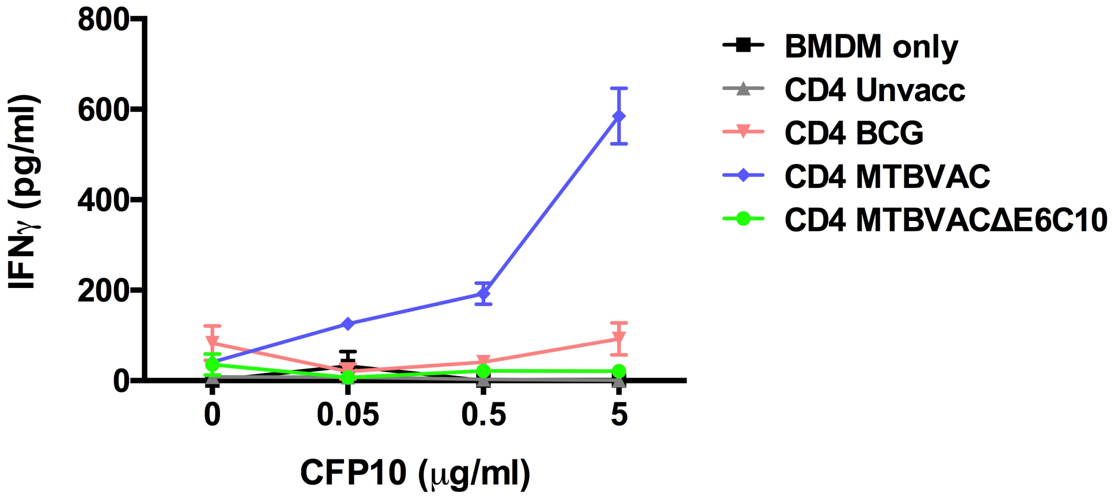

Supplementary Figure 5. *In vitro* CFP10 presentation assay. IFNγ production by purified CD4+ splenocytes from BCG-, MTBVAC- and MTBVACΔE6C10- vaccinated, or unvaccinated C3H/HeNRj mice following incubation with syngeneic BMDM previously pulsed with CFP10 increasing concentrations. Data represent the mean of three replicates ± SEM.

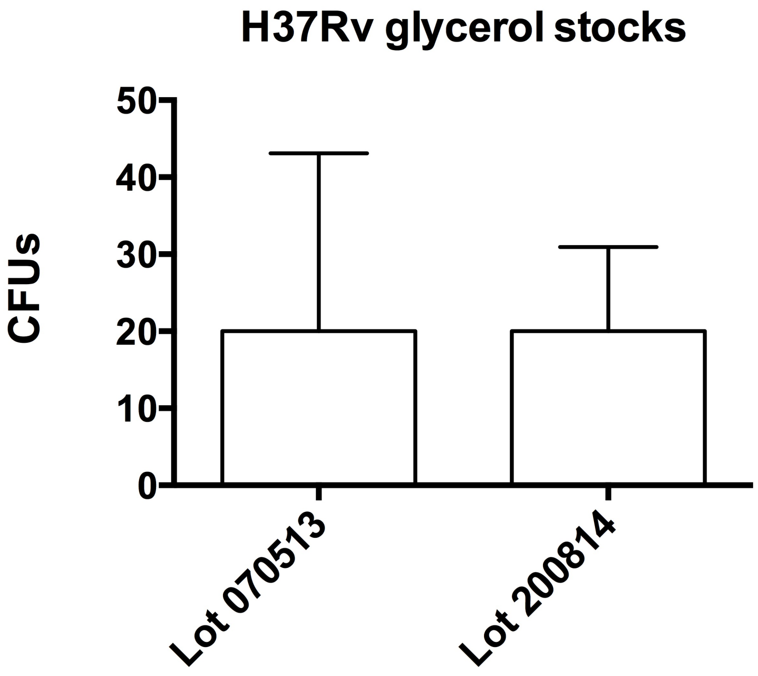

Supplementary Figure 6. Lung bacterial load one day post low-dose H37Rv intranasal challenge. Results correspond to the two H37Rv glycerol stocks used in the present study. Data are derived from n=10 mice (lot 070513) and n=6 mice (lot 200814). All data are mean ± SD.

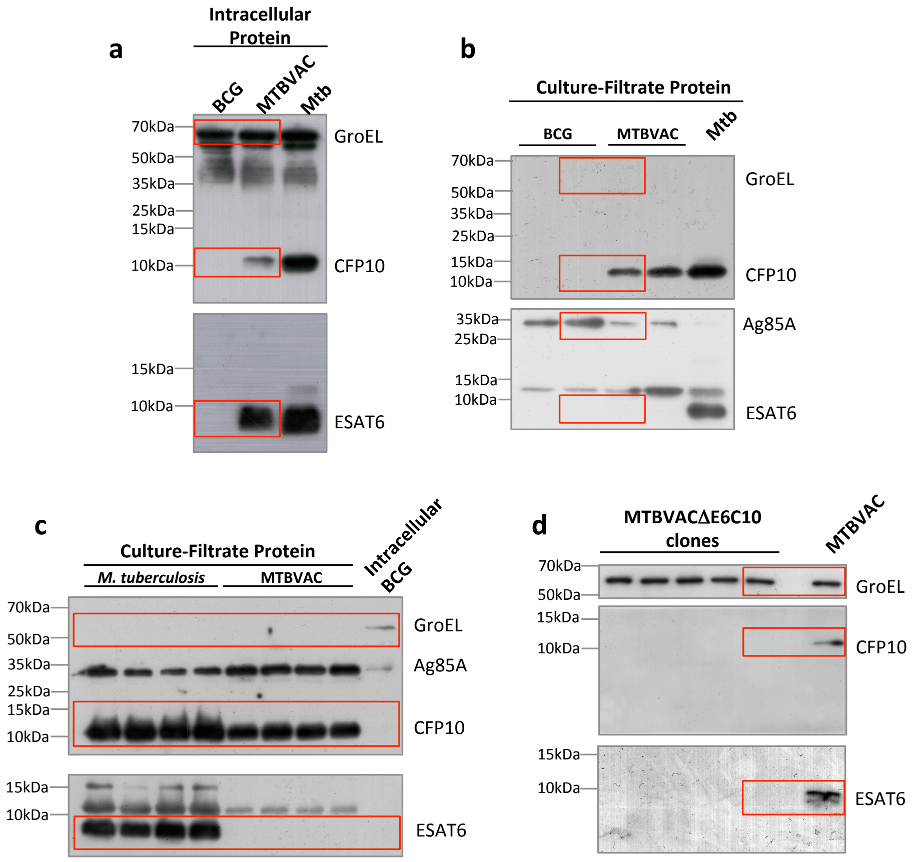

Supplementary Figure 7. Full western blots performed in this study. (a) Full blot corresponding to intracellular protein analysis from figure 1b. Blot was sequentially stained with anti-GroEL and anti-CFP10. Then membrane was incubated with a stripping solution and re-probed with anti-ESAT6. (b) Full blot corresponding to culture-filtrate protein fraction analysis from figure 1b. Blot was sequentially probed with anti-GroEL and anti-CFP10, and then re-probed with anti-Ag85A and anti-ESAT6 after stripping treatment. (c) Full blot corresponding to figure 1c. Blot was sequentially stained with anti-GroEL, anti-Ag85A and anti-CFP10, and then re-probed with anti- anti-ESAT6 after stripping treatment. (d) Full blot corresponding to figure 3a. Membrane was divided in two fragments. The upper one was probed with anti-GroEL, and the lower with anti-CFP10. Then, this was re-probed with anti-ESAT6 following stripping treatment. Red rectangles correspond to the blot fragments shown in the main figures.
